# The Presence of Anti-Lactoferrin Antibodies in a Subgroup of Eosinophilic Granulomatosis with Polyangiitis Patients and Their Possible Contribution to Enhancement of Neutrophil Extracellular Trap Formation

**DOI:** 10.3389/fimmu.2016.00636

**Published:** 2016-12-23

**Authors:** Haruki Shida, Daigo Nakazawa, Yu Tateyama, Arina Miyoshi, Yoshihiro Kusunoki, Fumihiko Hattanda, Sakiko Masuda, Utano Tomaru, Tamihiro Kawakami, Tatsuya Atsumi, Akihiro Ishizu

**Affiliations:** ^1^Division of Rheumatology, Endocrinology and Nephrology, Hokkaido University Graduate School of Medicine, Sapporo, Japan; ^2^Undergraduate School of Health Sciences, Hokkaido University, Sapporo, Japan; ^3^Department of Pathology, Hokkaido University Graduate School of Medicine, Sapporo, Japan; ^4^Department of Dermatology, St. Marianna University School of Medicine, Kawasaki, Japan; ^5^Faculty of Health Sciences, Hokkaido University, Sapporo, Japan

**Keywords:** lactoferrin, anti-lactoferrin antibody, neutrophil, neutrophil extracellular trap, eosinophilic granulomatosis with polyangiitis

## Abstract

Lactoferrin (Lf) is one of the antigens of antineutrophil cytoplasmic antibodies (ANCA) and functions as an endogenous suppressor of neutrophil extracellular trap (NET) formation. However, the prevalence and pathogenicity of anti-lactoferrin antibodies (aLf) in ANCA-associated vasculitis (AAV) remain unrevealed. This study aimed to examine the significance of aLf in AAV, initially. Sixty-five sera from AAV patients, including 41 microscopic polyangiitis, 5 granulomatosis with polyangiitis, and 19 eosinophilic granulomatosis with polyangiitis (EGPA) patients, were subjected to aLf detection using enzyme-linked immunosorbent assay. Clinical characteristics were compared between aLf-positive and aLf-negative patients. Neutrophils from healthy donors were exposed to suboptimal dose (10 nM) of phorbol myristate acetate (PMA) with aLf followed by evaluation of NET formation. Results demonstrated that 4 out of 65 AAV sera (6.2%) were positive for aLf. All of them were EGPA sera (4/19, 21.1%). In EGPA, the frequency of renal involvement, serum CRP levels, and Birmingham Vasculitis Activity Score (BVAS) in the aLf-positive patients was significantly higher than those in the aLf-negative patients, and the aLf titer correlated positively with the serum CRP level and BVAS. The NET formation was particularly enhanced by combined stimulation of 10 nM PMA and 1 µg/mL aLf. IgG isolated from sera of the aLf-positive EGPA patients (250 µg/mL) enhanced NET formation induced by 10 nM of PMA, and the effect was abolished completely by absorption of the aLf. This pilot study suggests that aLf enhance NET formation induced by PMA and are associated with disease activity of EGPA.

## Introduction

Antineutrophil cytoplasmic antibody (ANCA)-associated vasculitis (AAV) is characterized by pauci-immune necrotizing small vessel vasculitis with the presence of ANCA in the serum. AAV includes microscopic polyangiitis (MPA), granulomatosis with polyangiitis (GPA), and eosinophilic granulomatosis with polyangiitis (EGPA) (1). The major target antigens of ANCA are myeloperoxidase (MPO) and proteinase 3 (PR3). In MPA, the renal glomeruli are affected preferentially and the majority of the patients are positive for MPO–ANCA. Inflammation that is not centered on small vessels, including granulomatous inflammation, is generally absent. On the contrary, GPA displays necrotizing granulomatous inflammation that usually involves the respiratory tract and simultaneously develops necrotizing small vessel vasculitis. Pauci-immune type necrotizing crescentic glomerulonephritis is common in GPA, as well as in MPA. Typically, GPA patients are positive for PR3-ANCA. EGPA is an eosinophil-rich and necrotizing granulomatous vasculitis that affects predominantly small- to medium-sized vessels. This disease is associated with asthma or allergic sinusitis. The prominence of eosinophils in the blood and affected tissues is an essential feature of this disease. Approximately half of EGPA patients are positive for MPO–ANCA.

Lactoferrin (Lf) is present in specific granules of neutrophils (2). It may represent a target for ANCA in patients with autoimmune connective tissue diseases, such as arthritis and systemic lupus erythematosus (3, 4). Although earlier studies have demonstrated that lupus patients with anti-lactoferrin antibodies (aLf) exhibit higher disease activity than those without aLf (5, 6), the prevalence and pathogenicity of aLf in AAV remain unrevealed.

Lactoferrin is immediately secreted by degranulation upon activation of neutrophils (7). Recently, Okubo et al. have reported that Lf could play a role as an endogenous suppressor for neutrophil extracellular trap (NET) formation in activated and dying neutrophils (8). NETs are composed of extracellularly spreading chromatin fibers and neutrophil intracellular granule proteins, such as MPO and PR3 (9). Accumulating evidence indicates that excessive NET formation is involved in the pathogenesis of AAV (10–16).

In the present study, sera from AAV patients and healthy controls were subjected to aLf detection in order to examine the prevalence of aLf in AAV. We investigated correlations between the titers of aLf and clinical parameters. Furthermore, we determined the contribution of aLf to NET formation.

## Materials and Methods

### Patients and Serum Samples

In order to examine the prevalence and pathogenicity of aLf in AAV initially, 65 AAV patients including 41 MPA, 5 GPA, and 19 EGPA patients, who were diagnosed and treated at the Department of Internal Medicine II, Hokkaido University Hospital or Department of Dermatology, St. Marianna University Hospital from January 2005 to April 2014, were enrolled in this study. For controls, 10 healthy volunteers were included. Because this is a pilot exploratory study, a random study population size was chosen. After acquirement of written informed consent, peripheral blood was obtained without anticoagulants, and the sera were stored at −80°C until use. Clinical information, including age and gender, laboratory data, such as serum levels of MPO–ANCA and CRP, and Birmingham Vasculitis Activity Score (BVAS) of the AAV patients at the point of blood sampling were collected from medical records retrospectively. The history of asthma and eosinophil count in the peripheral blood were also collected concerning EGPA patients. This study was approved for practice by our Institutional Ethical Committee, the Ethical Committee of the Faculty of Health Sciences, Hokkaido University (Permission No. 15-90).

### Quantification of Anti-Lf Antibodies

Titer of aLf was determined using the enzyme-linked immunosorbent assay (ELISA) kit (Orgentec Diagnostika GmBH, Mainz, Germany).

### Isolation of Neutrophils

Human neutrophils were obtained from 20 mL of peripheral blood of healthy volunteers by density centrifugation using Polymorph Prep (Axis-Shield, Dundee, Scotland). After washing with PBS, the obtained cells were resuspended in RPMI 1640 medium supplemented with 5% fetal bovine serum.

### Reagents

For *in vitro* assay, rabbit polyclonal anti-human Lf antibodies (CSB-PA00870EORb) (Cusabio Biotech, Hubei, China) and rabbit control IgG (Beckman Coulter, Tokyo, Japan) were employed. Prior to use, the contaminating endotoxin was removed using ProteoSpin Endotoxin Removal Micro Kit (Norgentic Biotech, ON, Canada). Residual endotoxin was ruled out using Limulus Color KY Test Kit (Wako Pure Chemical, Osaka, Japan). Phorbol myristate acetate (PMA) was purchased from Sigma-Aldrich (St. Louis, MO, USA).

### NET Induction Assay

Peripheral blood neutrophils obtained from healthy volunteers were seeded on chamber slides (1 × 10^5^/mL), incubated for 15 min at 37°C, and then exposed to 0 or 10 nM PMA combined with 1 µg/mL aLf (CSB-PA00870EORb) or control rabbit IgG. After incubation for 3 h at 37°C, the samples were fixed with 4% paraformaldehyde (PFA) followed by mounting with the solution containing 4′,6-diamidino-2-phenylindole (DAPI) (Sigma-Aldrich). NET area was represented by DAPI-positive area, which was calculated using Image J software, as described previously (12, 17).

### Isolation of IgG from Serum

IgG was isolated from sera using an immunoadsorbent column (Protein G HP SpinTrap) (GE healthcare, Tokyo, Japan). Contamination by endotoxin in the IgG samples was ruled out using the endotoxin detection kit.

### Absorption of aLf in Serum

Recombinant human Lf (10 µg) (Hölzel Diagnostika, Cologne, Germany) was added to 500 µL patient sera. After incubation for 15 min at room temperature, IgG was purified using the immunoadsorbent column. The absorption of aLf was confirmed using WIESLAB ANCA Panel Kit (Euro Diagnostica, Malmö, Sweden). Residual Lf was confirmed as below the detection limit in Lf ELISA Kit (Assaypro, St. Charles, MO, USA).

### NET Induction by Patient IgG before and after Absorption of aLf

Peripheral blood neutrophils obtained from healthy volunteers were seeded on chamber slides (1 × 10^5^/mL), incubated for 15 min at 37°C, and then exposed to 0 or 10 nM PMA combined with 250 µg/mL patient IgG (before and after absorption of aLf) or healthy control IgG. After incubation for 3 h at 37°C, the samples were fixed with 4% PFA followed by mounting with a solution containing DAPI. NET area was represented by DAPI-positive area, which was calculated using Image J software.

### Statistical Analysis

Data of *in vitro* assay were obtained from experiments repeated five times and presented as mean ± SD values. Paired or unpaired Student’s *t*-tests, Mann–Whitney *U*-tests, one-way ANOVA, and Fisher’s exact test were applied appropriately for statistical evaluation with GraphPad Prism 5.0 software. *p*-values of less than 0.05 were regarded as statistically significant.

## Results

### Anti-Lf Antibodies in AAV Patients

**Figure 1:**
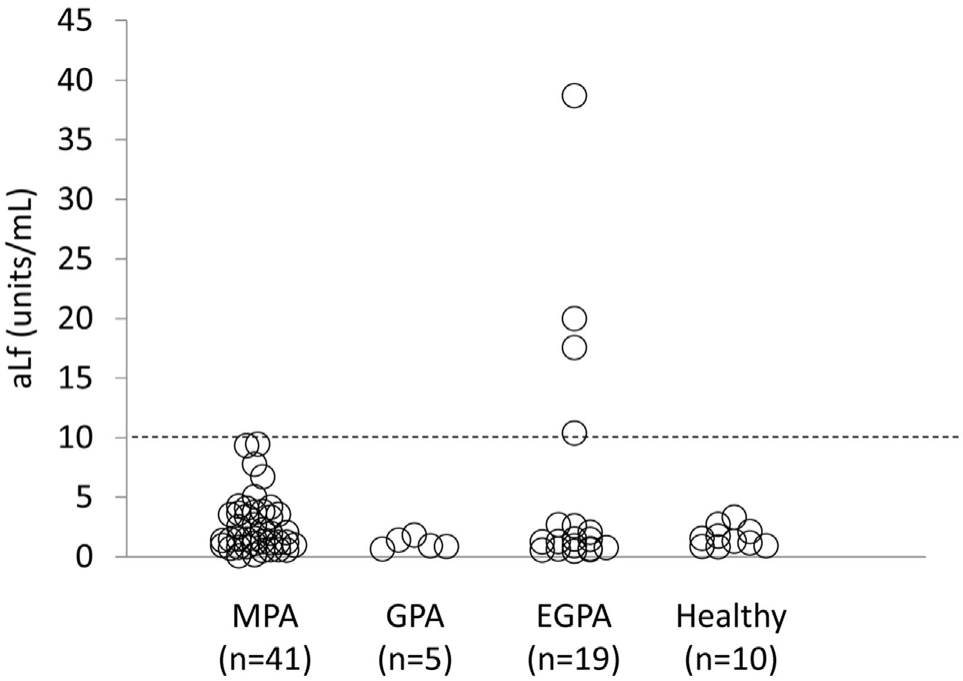
**Serum levels of aLf in anti-neutrophil cytoplasmic antibodies-associated vasculitis patients and healthy controls**. Titer of anti-lactoferrin antibodies was determined using the enzyme-linked immunosorbent assay kit. Cutoff value of the kit is represented by a broken line (10 units/mL).

Sixty-five AAV patients, including 41 MPA, 5 GPA, and 19 EGPA patients, were enrolled in this study. The demographics are summarized in Table 1. Serum titer of aLf was determined in the AAV patients (*n* = 65) and healthy volunteers (*n* = 10) (Figure 1). Cutoff value of the ELISA kit used for this assay is 10 units/mL. Four out of 65 AAV sera (6.2%) were positive for aLf, whereas none of the sera from healthy volunteers was aLf-positive (0%). All of the aLf-positive sera were obtained from EGPA patients. The aLf-positive rate in EGPA was 21.1% (4/19).

**Table 1:** **Demographics of antineutrophil cytoplasmic antibodies (ANCA)-associated vasculitis patients enrolled in this study**.

	Microscopic polyangiitis (*n* = 41)	Granulomatosis with polyangiitis (*n* = 5)	Eosinophilic granulomatosis with polyangiitis (*n* = 19)
Age, years, mean (SD)	69.5 (11.0)	47.8 (6.10)	54.6 (12.6)
Female, *n* (%)	24 (58.5%)	5 (100%)	13 (68.4%)
Myeloperoxidase–ANCA positive, *n* (%)	27 (65.9%)	0 (0%)	7 (36.8%)
CRP, mg/dL, mean (SD)	7.69 (5.63)	6.71 (3.94)	3.20 (4.19)
Birmingham Vasculitis Activity Score, mean (SD)	13.9 (8.03)	17.7 (9.12)	17.6 (11.4)

### Comparison of Clinical Characteristics of EGPA with or without aLf

**Figure 2:**
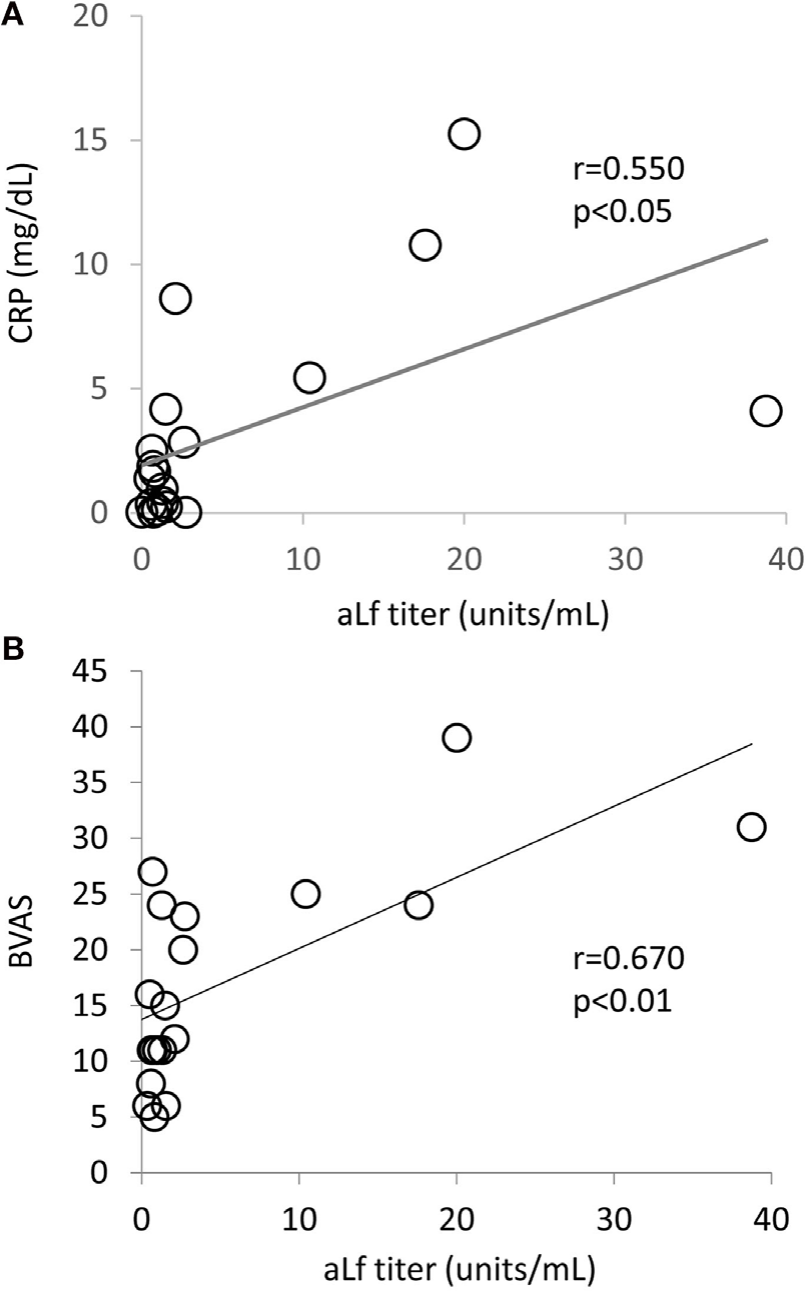
**Correlation of anti-lactoferrin antibodies titer with serum CRP level (A) and Birmingham Vasculitis Activity Score (B) in eosinophilic granulomatosis with polyangiitis patients (*n* = 19)**.

The differences in clinical characteristics of EGPA patients with or without aLf were determined. Although age, gender, history of asthma, eosinophil count in the peripheral blood, and positive rate of MPO–ANCA were equivalent among the EGPA patients regardless of the presence of aLf, the serum CRP levels and BVAS in the aLf-positive EGPA patients (*n* = 4) were significantly higher than those in the aLf-negative EGPA patients (*n* = 15) (Table 2). Correspondingly, the aLf titer showed positive correlation with the serum CRP level (*r* = 0.55, *p* < 0.05) and BVAS (*r* = 0.67, *p* < 0.01) (Figure 2). These findings indicate that the aLf titer is associated with disease activity of EGPA.

**Table 2:** **Comparison of clinical characteristics of eosinophilic granulomatosis with polyangiitis patients with or without aLf**.

	aLf-positive (*n* = 4)	aLf-negative (*n* = 15)	*p*-Value
Age, years, mean (SD)	56.6 (3.33)	54.0 (13.7)	n.s.^a^
Female, *n* (%)	3 (75.0%)	10 (66.7%)	n.s.^b^
Asthma, month, mean (SD)	27.8 (16.6)	42.6 (24.0)	n.s.^a^
Eosinophil count/μL, mean (SD)	12,500 (8,170)	7,420 (4,400)	n.s.^a^
MPO–ANCA positive, *n* (%)	3 (75.0%)	4 (26.7%)	n.s.^b^
CRP, mg/dL, mean (SD)	8.89 (4.44)	1.68 (0.96)	*p* < 0.001^a^
BVAS, mean (SD)	29.8 (5.97)	14.5 (12.0)	*p* < 0.01^a^

*^a^Student’s t-test*.

*^b^Fisher’s exact test*.

*n.s., not significant; BVAS, Birmingham Vasculitis Activity Score; aLf, anti-lactoferrin antibodies; MPO, myeloperoxidase; ANCA, anti-neutrophil cytoplasmic antibodies*.

### Anti-Lf Antibodies Enhance NET Formation Induced by PMA

**Figure 3:**
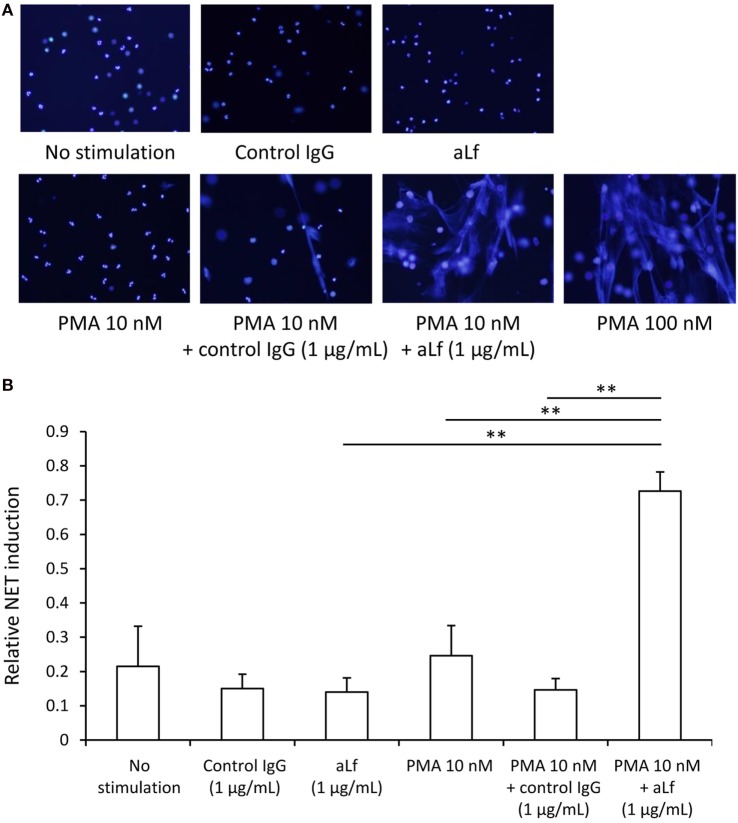
**NET induction assay**. Peripheral blood neutrophils obtained from healthy volunteers were seeded on chamber slides (1 × 10^5^/mL), incubated for 15 min at 37°C, and then exposed to 0 or 10 nM phorbol myristate acetate (PMA) combined with 1 µg/mL aLf (CSB-PA00870EORb) or control rabbit IgG. After incubation for 3 h at 37°C, the samples were fixed with 4% paraformaldehyde followed by mounting with a solution containing DAPI. For positive control, the neutrophils were exposed to 100 nM PMA for 3 h at 37°C. **(A)** The representative photomicrographs are shown (original magnification: ×200). **(B)** NET area was represented by DAPI-positive area, which was calculated using Image J software. Data were presented as mean ± SD values of relative NET induction in which the value of the positive control (PMA 100 nM) was set as 1. Experiments were repeated five times. **p < 0.01.

Since Lf was shown to function as an endogenous suppressor for NET formation, we hypothesized that aLf could interfere with the inhibitory role of Lf and result in enhancement of NET formation induced by PMA. Thus, the influence of aLf on NET formation was determined *in vitro*. Although aLf alone (1 µg/mL) did not induce NET formation in neutrophils (1 × 10^5^/mL), the aLf enhanced NET formation when combined with a suboptimal dose (10 nM) of PMA (Figure 3A). Repeated experiments demonstrated that the effect was statistically significant (Figure 3B).

### Anti-Lf Antibodies in EGPA Sera Enhance NET Formation Induced by PMA

**Figure 4:**
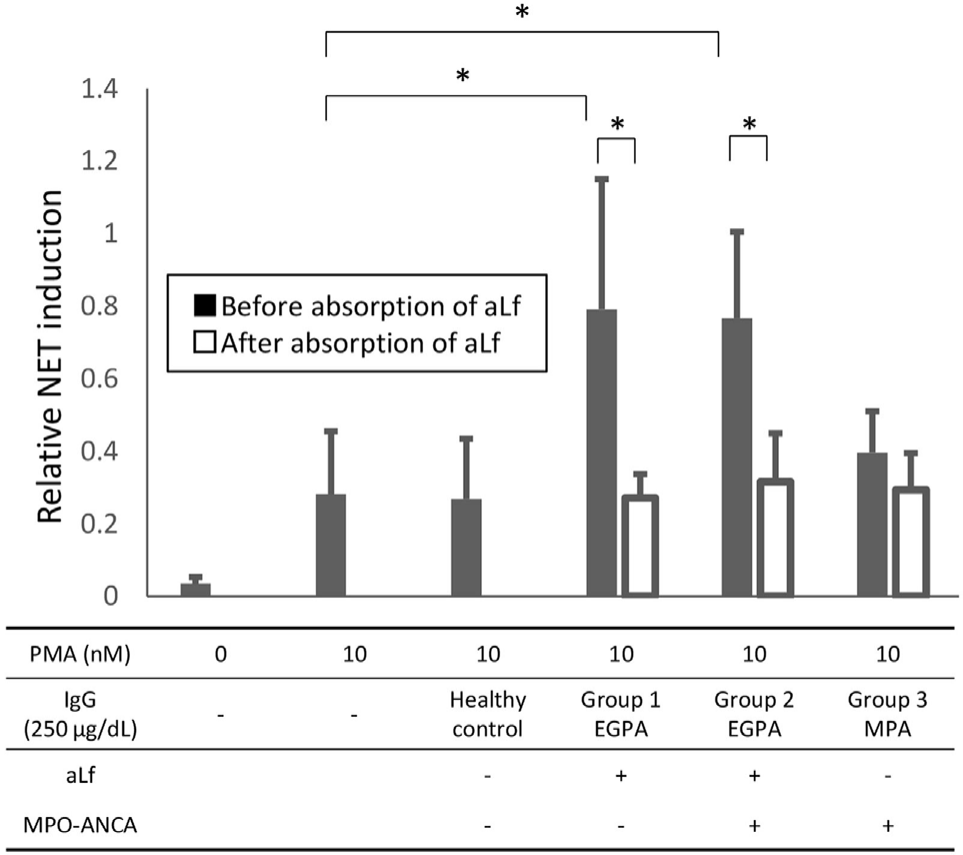
**Neutrophil extracellular trap (NET) induction by patient IgG before and after absorption of anti-lactoferrin antibodies (aLf)**. For this purpose, eosinophilic granulomatosis with polyangiitis sera with aLf were divided into the following two groups; Group 1, aLf-positive/myeloperoxidase (MPO)–anti-neutrophil cytoplasmic antibodies (ANCA)-negative (*n* = 1) and Group 2, aLf-positive/MPO–ANCA-positive (*n* = 3). Sera from a healthy volunteer and from microscopic polyangiitis patients (Group 3, aLf-negative/MPO–ANCA-positive, *n* = 3) were used as controls. Peripheral blood neutrophils obtained from healthy volunteers were seeded on chamber slides (1 × 10^5^/mL), incubated for 15 min at 37°C, and then exposed to 0 or 10 nM phorbol myristate acetate (PMA) combined with 250 µg/mL patient IgG (before and after absorption of aLf) or healthy control IgG. After incubation for 3 h at 37°C, the samples were fixed with 4% paraformaldehyde followed by mounting with the solution containing DAPI. For positive control, the neutrophils were exposed to 100 nM PMA for 3 h at 37°C. NET area was represented by DAPI-positive area, which was calculated using Image J software. Data were presented as mean ± SD values of relative NET induction in which the value of the positive control (PMA 100 nM) was set as 1. Experiments were repeated five times. *p < 0.05.

Next, we examined if aLf in EGPA sera could enhance NET formation induced by PMA as well. For this purpose, EGPA sera with aLf were divided into the following two groups; Group 1, aLf-positive/MPO–ANCA-negative (*n* = 1) and Group 2, aLf-positive/MPO–ANCA-positive (*n* = 3). Sera from a healthy volunteer and from MPA patients (Group 3, aLf-negative/MPO–ANCA-positive, *n* = 3) were used as controls. As shown in Figure 4, IgG isolated from EGPA sera with aLf (250 µg/mL) enhanced NET formation induced by the suboptimal dose (10 nM) of PMA regardless of the presence of MPO–ANCA (both in Group 1 and Group 2). Moreover, this effect was abolished completely by absorption of aLf using recombinant Lf (both in Group 1 and Group 2). On the contrary, IgG eluted from MPA sera with MPO–ANCA but without aLf (Group 3) did not exhibit the enhancement. The collective findings suggest that aLf in EGPA sera contribute to enhanced NET formation when some stimuli, which mimic PMA, act on neutrophils *in vivo*.

## Discussion

In the present study, we have demonstrated that some EGPA patients have aLf in the serum, and that the aLf titer is correlated with the disease activity. Furthermore, we have demonstrated that aLf (not only commercially available polyclonal antibodies but also those in EGPA sera) can enhance NET formation induced by a suboptimal dose of PMA *in vitro*. This is the first evidence that focuses on the significance of aLf in AAV, especially in EGPA.

The stimulation with aLf alone (1 µg/mL) did not induce NET formation in neutrophils (1 × 10^5^/mL). This finding is consistent with the presence of Lf in the specific granules of neutrophils under unstimulated conditions (2). On the contrary, the combined stimulation with aLf (1 µg/mL) and a suboptimal dose (10 nM) of PMA enhanced significantly *in vitro* NET formation. It has been shown that Lf was immediately secreted by degranulation upon activation of neutrophils (7). More recently, Okubo et al. demonstrated that Lf suppressed NET formation through charge-dependent interaction with the chromatin fibers in netting neutrophils (8). The Lf secreted by 10 nM PMA is considered to be sufficient for the inhibition of the NET induction force of 10 nM PMA so that the dose of PMA is suboptimal. We hypothesize that, when aLf is present, it could cancel out the endogenous suppressor effect of Lf, resulting in the enhancement of NET formation even under the suboptimal stimulation with PMA.

Another possibility of direct induction of NETs by aLf is also considered. It has been shown that Lf was expressed on the cell surface of primed neutrophils, and that aLf bound with cell surface Lf activated the neutrophils (18). Since the suboptimal dose of PMA (10 nM) induced cell surface expression of Lf (our unpublished results), aLf could activate neutrophils through interaction with the cell surface Lf resulting in enhancement of NET formation. Further studies are needed to reveal the mechanism of how aLf enhance the NET formation induced by a suboptimal dose of PMA.

Accumulating evidence indicates that excessive NET formation contributes directly to the dysfunction of vascular endothelial cells and results in the development of vasculopathy (19, 20). NET components, including MPO, which can mediate the generation of hypochlorite (OCl^−^) (21), histones (22), and matrix metalloproteinases (MMP), particularly MMP-2 and MMP-9 (23), have been demonstrated as strong effectors that induced vascular endothelial cell damage. In fact, the presence of extracellular MPO and NET-related molecules, such as histones, has been shown in the glomerular lesions of AAV (10, 24, 25). In addition, the increase in expressions of both MMP-2 and MMP-9 has been shown in the respiratory mucosa of EGPA patients (26). These reports correspond with our finding that the titer of aLf, which can contribute to enhancement of NET formation, is associated with the disease activity of EGPA.

Currently, it remains unrevealed which factors collaborate with aLf to induce NET formation in EGPA patients, as does PMA in the assay *in vitro*. Earlier studies have demonstrated that aLf could activate neutrophils primed by bacteria-derived f-Met–Leu–Phe (fMLP) (18), but not by C5a or calcium ionophore (27). In addition, fMLP was shown to induce NET formation in neutrophils (28). It has been well-known that preceding infection often triggers the onset and recurrence of AAV, including EGPA. Accordingly, bacteria-derived fMLP can be a candidate for ally of aLf to induce NET formation in EGPA patients.

Among the three AAV types, including MPA, GPA, and EGPA, aLf were detected in the EGPA sera exclusively in this study. A characteristic of EGPA is the presence of asthma or paranasal sinusitis history preceding the development of the disease. A recent study has demonstrated that a specific allergen for asthma induced Lf secretion from neutrophils derived from asthmatic patients (29). In asthma patients, however, ANCA, including aLf, are usually negative (30). Therefore, it is considered that the tolerance to Lf can be suspended with an undetermined reason resulting in the production of aLf in EGPA patients particularly. Although further studies are needed to clarify the mechanism of aLf production, the serum aLf can be a potential marker for the disease activity of some EGPA patients.

## Author Contributions

HS and YT carried out the experiments. HS, YK, AM, FH, and TK contributed to the collection of serum samples. HS, DN, SM, UT, TA, and AI analyzed the data. HS, UT, and AI designed the research and wrote the manuscript.

## Conflict of Interest Statement

The authors declare that the research was conducted in the absence of any commercial or financial relationships that could be construed as a potential conflict of interest.
